# Unilateral K-F ring in Wilson’s disease

**DOI:** 10.3205/oc000216

**Published:** 2023-03-01

**Authors:** Shruti P. Hegde, Sakthivel Senthil Kumar

**Affiliations:** 1 Manipal Tata Medical College, Manipal Academy of Higher Education, Jamshedpur, India; 2 Shri Sathya Sai Medical College and Research Institute, Ammapettai, India

**Keywords:** Kayser-Fleischer ring, unilateral, diagnosis, Wilson’s disease

## Abstract

Wilson’s disease, also called hepatolenticular degeneration, has varied clinical manifestations and poses diagnostic challenges. Kayser-Fleischer ring, when present, is considered pathognomic of Wilson’s disease. Although its presence is most commonly seen with the neuro-psychiatric form of the disease, it can also be present in hepatic form and asymptomatic patients. We report a case of unilateral Kayser-Fleischer ring in the normal, functional eye of a patient which subsequently led to the diagnosis of Wilson’s disease in the patient. This case also highlights the examination of the normal appearing eye in all the patients presenting with complaints in only one eye.

## Introduction

Wilson’s disease, the most common inborn error of copper metabolism, has varied clinical manifestations [1]. Due to this, it can pose serious diagnostic challenges. The main abnormality is mutation in the ATP7B gene causing a faulty copper excretion, thereby resulting in an abnormal copper deposition in liver and other organs. Deposition of copper in the cornea causes the Kayser-Fleischer ring (K-F ring). Unlike other places of abnormal deposition, K-F ring does not cause any functional abnormalities in the eye but is helpful in diagnosis and follow-up of the patient. Slit lamp examination, gonioscopy and anterior segment ocular coherence tomography are used for detection of K-F ring [2]. Due to the systemic nature of the disease, K-F rings are always bilateral. We report this interesting case of a unilateral K-F ring where the coincidental detection of K-F Ring in the normal eye led to the diagnosis of Wilson’s disease in the patient.

## Case description

**Figure 1:**
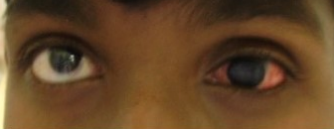
Patient with congestion of the left eye

**Figure 2:**
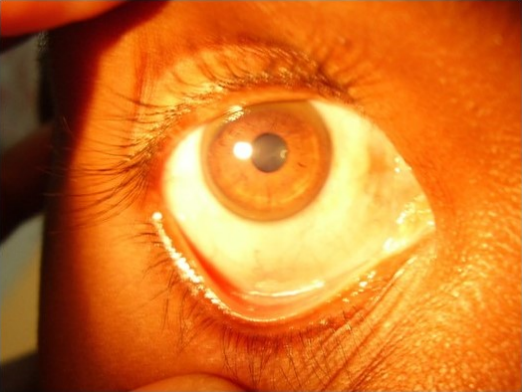
K-F ring in the right eye

A 35-year-old male patient of Indian origin visited our ophthalmic OPD with a history of redness in the left eye for one week (Figure 1 (Fig. 1)). He gave a history of cosmetic contact lens overwear in the same eye. The patient also had past history of childhood trauma and has had no vision in the left eye since childhood. On visual acuity testing, vision was 20/20 in the right eye and in the left eye perception of light was absent. Slit lamp examination showed diffuse conjunctival congestion and papillary hypertrophy of th upper palpebral conjunctiva and cosmetic contact lens over the cornea with proteinaceous aggregates over the lens. On removal of the lens, total leucomatous corneal opacity was found and further details could not be made out. Digital intraocular pressure (IOP) was reduced indicating that the eye was phithisical. A diagnosis of contact lens induced giant papillary conjunctivitis with corneal opacity with phthisis bulbi was made for the left eye. As a routine the right eye was examined, which showed the presence of a brownish coloured ring in the peripheral cornea at the level of Descemet’s membrane, which is classical of K-F ring (Figure 2 (Fig. 2)). The rest of the anterior segment and fundus examination was normal. 

On retrospective history taking, the patient did give history of neurological abnormalities including gradual deterioration of speech, gait and choreoathetoid movements for the past few months. The patient had visited multiple general practitioners in the past and was also referred to a psychiatrist for presumed behavioral abnormalities. The presence of a classical picture of K-F ring in the right eye along with the neurological abnormalities helped us in making a provisional diagnosis of Wilson’s disease. The patient was treated for allergic conjunctivitis in the left eye and sent back to a physician with a requisition for detailed evaluation. Patient’s MRI showed T2 hyperintensity in the putamen, deep gray nuclei, mid brain tegmentum and pons suggestive of Wilson’s disease. The diagnosis of Wilson’s disease was confirmed by a combination of low serum ceruloplasmin and high urine copper. He was initially put on d-Penicillamine, but his neurological symptoms worsened. So, he was shifted to tablet Zinc 50 mg once daily. Although he did not show drastic improvement, his symptoms plateaued and he was able to carry on with his daily routine.

## Discussion

K-F ring, by definition, is bilateral. Except for one case report, there has been no mention of unilateral K-F ring in the literature [3]. In the previous case of the unilateral K-F ring reported by Innes et al., one eye of the patient had been phithisical since childhood and K-F ring was absent in that eye. In comparison, our patient also had history of childhood trauma which resulted in corneal opacity as well as phthisis and non-functional eye. The source of copper that led to the formation of K-F ring has been debated to be either the limbal capillaries or the aqueous humor. The latter is the most widely accepted mechanism [4]. The absence of K-F ring in the phithisical eye in our patient as well as the previous case substantiates aqueous humor as the source of copper deposition. Copper is deposited as a granular complex with sulfur, which gives the ring its characteristic color [5]. It is to be noted that copper is present throughout the cornea, however, due to fluid streaming, copper tends to accumulate superiorly and inferiorly before involving the cornea circumferentially. The direction of aqueous flow and the functional peculiarity of the peripheral part of the corneal endothelial layer are also responsible for the peripheral location of the ring [6]. While in our patient the exact cause of unilaterality cannot be ascertained, the authors can offer a plausible explanation. Firstly, the other eye being phthisical, aqueous outflow is absent owing to hypoplasia and apoptosis of uveal tissues. Secondly, the deep extent of leucomatous corneal scarring might have prevented typical accumulation of copper granules. It is thus difficult to pinpoint why the K-F ring was absent in the non-functional eye as there was superadded corneal opacity.

Our case is interesting because the patient presented for complaints in the left eye, which did not show the presence of K-F ring. It was a routine examination of the normal right eye that led to the discovery of K-F ring. Apart from being the second case report of unilateral K-F ring, this case also highlights the importance of examination of the normal eye in all ophthalmic patients. The coincidental discovery of K-F ring in the normal eye in this patient led to the diagnosis of Wilson’s disease. 

## Notes

### Competing interests

The authors declare that they have no competing interests.
